# Estrogens increase expression of bone morphogenetic protein 8b in brown adipose tissue of mice

**DOI:** 10.1186/s13293-015-0025-y

**Published:** 2015-04-03

**Authors:** Aldo Grefhorst, Johanna C van den Beukel, E Leonie AF van Houten, Jacobie Steenbergen, Jenny A Visser, Axel PN Themmen

**Affiliations:** Department of Internal Medicine, Erasmus MC, University Medical Center Rotterdam, Room Ee532, P.O. Box 2040, 3000 CA Rotterdam, The Netherlands

**Keywords:** BMP8b, Brown adipose tissue, Diethylstilbestrol (DES), FGF1, UCP1

## Abstract

**Background:**

In mammals, white adipose tissue (WAT) stores fat and brown adipose tissue (BAT) dissipates fat to produce heat. Several studies showed that females have more active BAT. Members of the bone morphogenetic protein (BMP) and fibroblast growth factor (FGF) families are expressed in BAT and are involved in BAT activity. We hypothesized that differential expression of BMPs and FGFs might contribute to sex differences in BAT activity.

**Methods:**

We investigated the expression of BMPs and FGFs in BAT of male and female C57BL/6J mice upon gonadectomy, cold exposure, and exposure to sex steroids.

**Results:**

Of the FGF family, BAT *Fgf1*, *Fgf9*, *Fgf18*, and *Fgf21* expression was induced upon cold exposure, but only *Fgf1* expression was obviously different between the sexes: females had 2.5-fold lower BAT *Fgf1* than males. Cold exposure induced BAT *Bmp4* and *Bmp8b* expression, but only *Bmp8b* differed between the sexes: females had 35-fold higher BAT *Bmp8b* than males. Ovariectomy almost completely blunted BAT *Bmp8b* expression, while orchidectomy had no effect. Male mice and ovariectomized female mice treated with diethylstilbestrol (DES) had approximately 350-fold and approximately 36-fold higher BAT *Bmp8b* expression, respectively. Ninety-day and 7-day treatment of female mice with dihydrotestosterone (DHT) decreased BAT *Bmp8b* expression by approximately fivefold and approximately fourfold, respectively. Finally, treatment of primary murine brown adipocytes with DES did not result in changes in *Bmp8b* expression.

**Conclusions:**

BAT *Bmp8b* expression in mice is positively regulated by presence of ovaries and estrogens such as DES.

## Background

Adipose tissue is an important mediator of energy balance in mammals. White adipose tissue (WAT) stores energy in the form of energy dense triglycerides (TGs) while brown adipose tissue (BAT) has the unique ability to oxidize fatty acids released from TGs to generate heat, a process termed thermogenesis [1]. The mitochondrial uncoupling protein 1 (UCP1) is predominantly expressed in BAT and controls the thermogenetic properties of this tissue. UCP1 uncouples ATP synthesis from oxidative phosphorylation in the mitochondria, a process that generates heat. Cold exposure of adult humans causes enhanced ^18^F-deoxyglucose (^18^F-FDG) uptake in the upper chest and neck regions [2-4], showing that BAT is present and active in adult humans.

Animal experiments have shown that females have more active BAT than males [5-7]. For instance, BAT from female rodents contains more and bigger mitochondria [5] and has a higher lipolytic activity upon caloric restriction [6]. More importantly, human studies also show that, compared to men, women have more often ^18^F-DFG uptake in areas considered to contain BAT as determined by positron emission tomography (PET) scans [2,8-10]. A recent study confirmed that the relative contribution of fat mass to resting metabolic rate and the metabolic rate per kilogram adipose tissue were both higher in women than in men [11]. Furthermore, tissue gene expression of genes involved in mitochondrial function suggested that women have an increased number of brown adipocytes [11]. It is not entirely clear how this sex difference is regulated but certain aspects have been elucidated. The sex steroid hormone estradiol (E2) may be one of the regulators. Pedersen et al*.* [12], for instance, showed that treatment of ovariectomized rats with 17-β-estradiol pellets prevented ovariectomy-mediated reduction of BAT *Ucp1* mRNA expression. In cultured primary mouse brown adipocytes, E2 suppressed transcription of the α_2_-adrenergic receptor that inhibits rather than elevates cAMP upon norepinephrine (NE) activation [13] while testosterone suppressed *Ucp1* expression [14]. Presumably, the most important activator of UCP1 and BAT is sympathetic innervation [1], and Martínez de Morentin et al*.* [15] recently showed that E2 acts in the central nervous system to regulate BAT thermogenesis, specifically in the ventromedial nuclei of the hypothalamus.

A number of additional BAT activators such as thyroid hormone [1] have been discovered in recent years, but the hunt for new physiological relevant BAT activators continues, since autocrine and/or paracrine hormones or growth factors that activate the BAT depot may be considered novel candidates that can be used in treatment modalities to combat obesity. Two classes of paracrine/autocrine hormones or growth factors might be of particular interest given their role in brown adipocyte differentiation and function: bone morphogenetic proteins (BMPs) and fibroblast growth factors (FGFs). BAT expresses several BMP family members [16], among which BMP7 [17,18] and BMP8b [19] have been shown to activate BAT directly. Compared to their wild-type littermates, *Bmp7*^*−/−*^ mice have reduced amounts of BAT [18] and *Bmp8b*^*−/−*^ mice have impaired thermogenesis and reduced metabolic rate [19]. In addition, BMP7 induces brown preadipocyte differentiation [18]. Likewise, BAT expresses several FGF family members [20]. FGF21 is secreted by activated BAT [21,22], and both FGF1 and FGF2 have been shown to upregulate UCP1 expression in cultured rat brown adipocytes [23].

In the present studies, we investigated the differential expression of BMP and FGF family members in BAT of male and female mice. We therefore determined the mRNA expression of the most abundant BMP and FGF family members in male and female BAT upon cold exposure, gonadectomy, and sex steroid treatment. In short, we found that BMP8b might be one of the factors involved in sex-specific differences in BAT activity since BAT *Bmp8b* expression was higher in female mice than in male mice. Moreover, BAT *Bmp8b* expression in female mice was reduced by ovariectomy and induced by treatment of male mice and ovariectomized female mice with diethylstilbestrol (DES).

## Methods

### Animals

Male and female C57Bl/6J mice were obtained from Charles River Laboratories (Maastricht, The Netherlands) at the indicated age and were kept 1 week under standard housing conditions before they were enrolled in an experimental setup.

In the first experiment, 9-week-old male mice were individually housed in a temperature-controlled climate chamber (Bronson, Nieuwkuijk, The Netherlands) with normal light/dark cycle at 23°C or 4°C for 24 hours (*n* = 6 mice per group). After these 24 h, the mice were terminated by cardiac puncture under isoflurane anesthesia.

In the second and third experiment, 9-week-old male and female mice underwent gonadectomy or a sham operation under isoflurane anesthesia (*n* = 10 mice per group). For the female mice, gonadectomy involved a small incision in both flanks after which the ovaries were removed. In the male mice, small incisions were made in the lower abdomen through which the testes were removed. Sham-operated animals underwent the same procedures without removal of ovaries or testes. After the surgery, the mice were allowed to recover for 45 days. For the second experiment, mice were fasted for 4 h and terminated by cardiac puncture under isoflurane anesthesia. For the third experiment, the mice were put in the climate chamber for 24 h at 23°C or 4°C after which they were terminated by cardiac puncture under isoflurane anesthesia (*n* = 4 to 6 mice per group). Uterus weight of female mice in both experiments was measured to determine whether ovariectomy was successful.

For the fourth experiment, 9-week-old male mice received daily subcutaneous injections with 100 μg/kg DES (Steraloids Inc., Newport, RI) dissolved in olive oil or the olive oil vehicle alone for 1 week before they were terminated by cardiac puncture under isoflurane anesthesia (*n* = 6 mice per group).

For the fifth experiment, 9-week-old female mice underwent gonadectomy or a sham operation as described above (*n* = 6 mice per group). After 1 week of recovery, these mice received daily subcutaneous injections with 100 μg/kg DES dissolved in olive oil or the olive oil vehicle alone for 1 week before they were terminated by cardiac puncture under isoflurane anesthesia.

For the sixth experiment, 9-week-old female mice received daily subcutaneous injections with 100 μg/animal dihydrotestosterone (DHT) (Steraloids Inc.) dissolved in olive oil or the olive oil vehicle alone for 1 week before they were terminated by cardiac puncture under isoflurane anesthesia after which the uterus was weighed (*n* = 6 mice per group).

For the seventh experiment, 19-day-old female mice received a DHT or placebo pellet (Innovative Research of America, Sarasota, FL) as described previously [24] (*n* = 6 mice per group). After 90 days, the mice were terminated by decapitation under isoflurane anesthesia.

For all experiments, the intrascapular BAT depot was collected and stored at −80°C until analysis. All animal experiments were performed with the Approval of the Animal Ethics Committee at Erasmus MC, Rotterdam, The Netherlands.

### Primary cell cultures

The intrascapular BAT depot was harvested from 18 male mice that were terminated by cardiac puncture under isoflurane anesthesia. The depots were kept in ice-cold PBS until they were minced into small pieces. The pieces were digested with 0.1% m/v collagenase (Sigma-Aldrich, Zwijndrecht, The Netherlands), 0.1% m/v dispase II (Roche Diagnostics, Mannheim, Germany), and 0.05% m/v trypsin (Sigma-Aldrich) in serum-free culture medium (DMEM with 4.5 g/l D-glucose supplemented with antibiotic-antimyotic (Gibco, Bleiswijk, The Netherlands)) for 45 min at 37°C with gentle agitation. The enzymes were inactivated with an equal volume of culture medium with 10% FCS (Gibco) after which the samples were filtered through a 100-μm mesh filter to remove debris and spun down for 8 min at 1,200 rpm. The pellets were resuspended in RBC Lysis buffer (eBioscience, San Diego, CA) and lysed for 5 min after which the samples were spun down for 5 min at 1,500 rpm. The pellet was resuspended in culture medium with 10% FCS, the amount of viable cells counted and plated with 300,000 alive cells in conventional 24-well plates and cultured at 37°C with 5% CO_2_. Twenty-four hours later, the medium was replaced with differentiation medium: DMEM with 4.5 g/l D-glucose supplemented with 10% FCS, antibiotic-antimyotic, 4 nM bovine insulin (Sigma-Aldrich), 10 mM HEPES (Gibco), 4 mM glutamine (Gibco), 25 μg/ml ascorbate (Sigma-Aldrich), and 1 μM rosiglitazone (ENZO Lifesciences, Raamsdonkveer, The Netherlands). This differentiation medium was replaced every 2 or 3 days. After 12 days of differentiation, the medium was replaced with culture medium with 10% charcoal-stripped FCS (Gibco). After 8 h, this medium was replaced with culture medium with charcoal-stripped FCS supplemented with or without 10 μM DES. The cells were harvested and stored at −80°C until RNA isolation 24 h later.

### RNA isolation, cDNA synthesis, and real-time PCR

Total RNA from mouse tissues and cultured cells was isolated using Tripure Isolation Reagent (Roche) according to the manufacturer’s instructions. Genomic DNA was removed by DNAse treatment (Promega Benelux BV, Leiden, The Netherlands) for 30 min at 37°C. Reverse transcription was performed using a cDNA synthesis kit (Roche) according to the manufacturer’s instructions. Quantitative RT-PCR was performed using SYBRgreen mastermix (Applied Biosystems, Nieuwerkerk a/d IJssel, The Netherlands) with an ABI Prism 7900 Sequence Detection System. Sequences of the primers used are listed in Table 1. The expression of each gene was expressed in arbitrary units after normalization to the average expression level of the housekeeping genes 18S and beta-2 microglobulin using the 2^−ΔΔCt^ method [25].

**Table 1:** **Primer sequences**

**Gene**	**Accession no.**	**Forward primer**	**Reverse primer**	**Reference**
Rn18s	NR_003278	GTAACCCGTTGAACCCCATT	CCATCCAATCGGTAGTAGCG	
B2m	NM_009735	ATCCAAATGCTGAAGAACGG	CAGTCTCAGTGGGGGTGAAT	
Hprt	NM_013556	GCAGTACAGCCCCAAAAT	AACAAAGTCTGGCCTGTATCCAA	
Ucp1	NM_009463	GGCCTCTACGACTCAGTCCA	TAAGCCGGCTGAGATCTTGT	[47]
Bmp2	NM_007553	TGCTTCTTAGACGGACTGCG	CTGGGGAAGCAGCAACACTA	
Bmp4	NM_007554	GAGCCATTCCGTAGTGCCAT	AACGACCATCAGCATTCGGT	
Bmp5	NM_007555	ATGCCACCAACCATGCCATA	GCCACACGAACGTACTACCA	
Bmp6	NM_007556	AACGCACACATGAATGCCAC	CCACAAGCTCTCACGACCAT	
Bmp7	NM_007557	CGGGAGTTCCGGTTTGATCT	GCAAGAAGAGGTCCGACTCC	
Bmp8b	NM_007559	CAACCACGCCACTATGCAG	CACTCAGCTCAGTAGGCACA	
Fgf1	NM_010197	GAAGCATGCGGAGAAGAACTG	CGAGGACCGCGCTTACA	[48]
Fgf2	NM_008006	CAACCGGTACCTTGCTATGA	TCCGTGACCGGTAAGTATTG	[20]
Fgf6	NM_010204	ACACACGAGGAGAACCCCTA	TGGAAGCTGGGCGTTGTGTA	
Fgf9	NM_013518	TATCCAGGGAACCAGGAAAGAC	CAGGCCCACTGCTATACTGATAAA	[48]
Fgf10	NM_008002	GCGGGACCAAGAATGAAGA	AGTTGCTGTTGATGGCTTTGA	[20]
Fgf11	NM_010198	TTGTACAGCTCGCCACATTTC	GTAATTCTCAAAGACGCACTCCTT	[20]
Fgf13	NM_010200	AATGAACAGCGAGGGATACTTG	ACTGATTCTTTGAATTTGCACTCA	[20]
Fgf14	NM_207667	ACCCATCAGAACTTTTTACCCCT	ACATGGCAACTTCCAATGGCT	
Fgf16	NM_030614	GGCCTGTACCTAGGAATGAATGA	TTCCCGGAAAACACATTCAC	[20]
Fgf18	NM_008005	CATTCAAGTCCTGGGCCGTA	ATGAACACGCACTCCTTGCT	
Fgf21	NM_020013	CTGGGGGTCTACCAAGCATA	CACCCAGGATTTGAATGACC	[49]
Pparγ	NM_001127330	CAAGAATACCAAAGTGCGATCAA	GAGCTGGGTCTTTTCAGAATAATAAG	

### Immunoblot analysis

The murine intrascapular BAT depot was lysed in cold PBS supplemented with Phosphatase Inhibitor Cocktail 2 (Sigma-Aldrich) and cOmplete Protease Inhibitor Cocktail Tablets (Roche) followed by sonification for 10 s. The protein concentrations were determined using the BCA Protein Assay kit (Pierce, Rockford, IL). Individual samples were mixed with Laemmli loading buffer (Pierce), heated for 5 min at 96°C, and subjected to SDS-PAGE on a 10% gel. UCP1 was determined using a polyclonal anti-UCP1 antibody raised in rabbit (Sigma-Aldrich). As loading controls, the concentrations of α-tubulin was determined using a polyclonal anti-α-tubulin antibody raised in rabbit (Santacruz, Heidelberg, Germany). Finally, goat-anti-rabbit IRDye 800 secondary antibody (Li-cor, Leusden, The Netherlands) and an Odyssey fluorescence scanner (Li-cor) were used. The immunoblots were analyzed with Odyssey software.

### Statistical analysis

Statistics were performed with GraphPad Prism (GraphPad Software, Inc.). When 4°C was compared with 23°C, a Mann–Whitney *U* test was performed (*p* < 0.05 was considered significant). When the effect of sex and gonadectomy or sex and treatment was studied, a two-way ANOVA was performed. *Post hoc* Mann–Whitney *U* tests were performed (*p* < 0.025 being considered significant) when a significance (*p* < 0.05) was found in the two-way ANOVA.

## Results

### Multiple BMPs and FGFs are induced in BAT upon cold exposure

**Figure 1:**
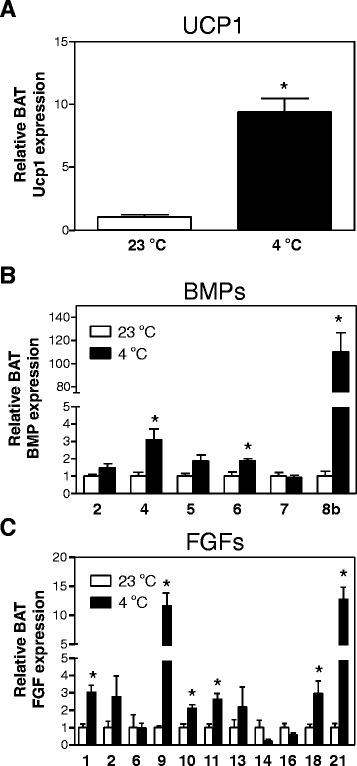
**Induction of three BMP family members and seven FGF family members upon cold exposure. (A)** *Ucp1* mRNA expression in BAT of male mice kept at 23°C or 4°C for 24 h. **(B)** Expression of genes encoding for BMP family members in BAT of male mice kept at 23°C or 4°C for 24 h. **(C)** Expression of genes encoding for FGF family members in BAT of male mice kept at 23°C or 4°C for 24 h. Results were normalized to 18S ribosomal RNA (*Rn18s*) and beta-2 microglobulin (*B2m*) with data from mice kept at 23°C defined as ‘1’.Values are averages ± SEM; *n* = 6; *p < 0.05 vs*.* 23°C (Mann–Whitney *U* test).

BMPs and FGFs are proteins with autocrine and/or paracrine actions of which many are expressed in BAT, as previously described [18,20,26]. We determined which of these BMP and FGF family members are associated with activated BAT. For this, male mice were exposed to 4°C for 24 h Exposure to 4°C resulted in a significantly stronger body weight loss compared to exposure to 23°C (1.87 ± 0.24 vs*.* 0.30 ± 0.09 gram lost during 24 h, 4°C vs*.* 23°C, *p* < 0.05). Cold exposure indeed activated BAT since *Ucp1* expression was almost tenfold upregulated (Figure 1A). Of the BMP family members, only expression of *Bmp4*, *Bmp6*, and *Bmp8b* was significantly upregulated in BAT upon cold exposure by 3.08 ± 0.64-, 1.89 ± 0.11-, and 110.06 ± 16.67-fold, respectively (*p* < 0.05) (Figure 1B). For the FGFs, cold exposure significantly increased *Fgf1*, *Fgf9*, *Fgf10*, *Fgf11*, *Fgf18*, and *Fgf21* expression by 3.02 ± 0.41-, 11.60 ± 2.21-, 2.12 ± 0.20-, 2.63 ± 0.34-, 2.97 ± 0.71-, and 12.78 ± 2.05-fold, respectively (*p* < 0.05) (Figure 1C). Since we aimed to study the link between BAT activity and BMPs and FGFs in relation to sex differences, the expression of these three BMP family members and six FGF family members was further analyzed in subsequent experiments.

### Female mice have higher BAT Bmp8b and lower BAT Fgf1 expression

**Figure 2:**
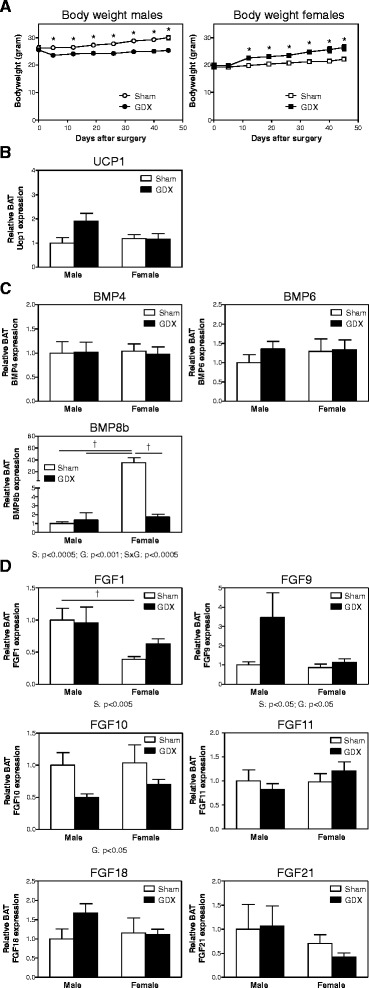
**Female mice have higher BAT** ***Bmp8b*** **expression and lower BAT** ***Fgf1*** **expression. (A)** Body weight of male and female mice following received GDX or a sham operation. **(B)** *Ucp1* mRNA expression in BAT of male and female mice upon GDX or sham operation. **(C)** *Bmp4*, *Bmp6*, and *Bmp8b* mRNA expression in BAT of male and female mice upon GDX or sham operation. **(D)** *Fgf1*, *Fgf9*, *Fgf10*, *Fgf11*, *Fgf18*, and *Fgf21* mRNA expression in BAT of male and female mice upon GDX or sham operation. mRNA expression results were normalized to 18S ribosomal RNA (*Rn18s*) and beta-2 microglobulin (*B2m*) with data from sham-operated male mice defined as ‘1’. Values are averages ± SEM; *n* = 10; †*p* < 0.025 (Mann–Whitney *U post hoc* test when a significance was detected by two-way ANOVA). Depicted below the graphs are the significant *p* values of the two-way ANOVA tests for either sex (S), GDX (G), or the interaction between sex and GDX (SxG).

Next, to determine whether BMP and FGF family members associated with activated BAT are differentially expressed in male and female BAT, we compared the expression of the three BMPs and six FGFs in the BAT depot of male and female mice. In addition, we determined whether gonadectomy (GDX) had an effect on their expression levels in order to investigate the contribution of gonadal function to the regulation of BAT activity. In male mice, GDX resulted in a reduced body weight while female mice gained weight when GDX’d (Figure 2A). Remarkably, BAT *Ucp1* mRNA expression did not differ between male and female mice (Figure 2B). Of the three BMP family members tested, only BAT *Bmp8b* expression showed a striking difference between the sexes (Figure 2C). Sham-operated female mice had a 35.4 ± 8.3-fold higher BAT *Bmp8b* expression compared to sham-operated male mice (*p* < 0.025) which was significantly reduced to levels observed in male mice upon ovariectomy. In contrast, orchidectomy had no effect on BAT *Bmp8b* expression. Of the six FGF family members, BAT *Fgf1* expression showed the largest significant difference between male and female mice (Figure 2D). Sham-operated female mice had lower BAT *Fgf1* expression than sham-operated male mice and ovariectomy tended to increase BAT *Fgf1* expression; however, this failed to reach significance. BAT *Fgf9*, *Fgf11*, *Fgf18*, and *Fgf21* mRNA expression was not significantly different between the four experimental groups. Finally, BAT *Fgf10* expression was affected by GDX, independent of sex

### Cold induces BAT *Bmp8b* and *Fgf1* expression, independent of sex or presence of gonads

**Figure 3:**
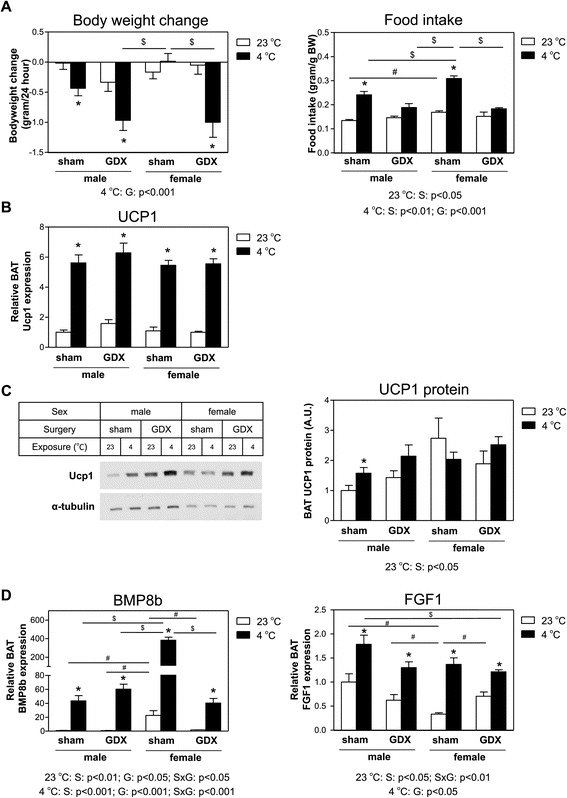
**Independent of sex and presence of gonads, cold exposure induces BAT** ***Fgf1*** **and** ***Bmp8b*** **expression. (A)** Change in body weight and food intake of sham-operated and GDX’d male and female mice during exposure to 4°C or 23°C for 24 h. **(B)** *Ucp1* mRNA expression in BAT of male and female mice upon GDX or sham operation and exposure to 4°C or 23°C for 24 h. **(C)** Immunoblot of UCP1 and α-tubulin and quantification of UCP1 protein vs. α-tubulin protein content in BAT of male and female mice upon GDX or sham operation and exposure to 4°C or 23°C for 24 h. UCP1 protein results were normalized with data from sham-operated male mice exposed to 23°C defined as ‘1’, *n* = 4. **(D)** *Bmp8b* and *Fgf1* mRNA expression in BAT of male and female mice upon GDX or sham operation and exposure to 4°C or 23°C for 24 h. mRNA expression results were normalized to 18S ribosomal RNA (*Rn18s*) and beta-2 microglobulin (*B2m*) with data from sham-operated male mice exposed to 23°C defined as ‘1’. Values are averages ± SEM; *n* = 4–6; *p < 0.05 4°C vs*.* 23°C for the same animal model (Mann–Whitney *U* test); #*p* < 0.025 (Mann–Whitney *U post hoc* test when significance was detected by two-way ANOVA for mice exposed to 23°C); $*p* < 0.025 (Mann–Whitney *U post hoc* test when significance was detected by two-way ANOVA for mice exposed to 4°C). Depicted below the graphs are the significant *p* values of the two-way ANOVA tests for either (S), GDX (G), or the interaction between sex and GDX (SxG) for mice exposed to either 23°C or 4°C.

The data so far show that of the genes studied, expression of *Bmp8b* and *Ffg1* in BAT differs between the sexes. Since both genes are also upregulated upon cold exposure, we next investigated what the effect of cold exposure on these two genes was in mice of both sexes combined with GDX. The effect of GDX on body weight of the male and female mice was comparable to that observed in the previous experiment (data not shown). Cold exposure enhanced metabolism as is evident from the effects of the 24-h exposure to 4°C on body weight and food intake (Figure 3A). The 4°C sham-operated mice ate approximately 50% more than those kept at 23°C, and this resulted in marginal effects on body weight. However, GDX’d mice did not eat more at 4°C compared to 23°C, and this resulted in a marked body weight loss of 1 g/24 h. Cold exposure induced BAT *Ucp1* mRNA expression up to sixfold (Figure 3B), but UCP1 protein content was only marginally induced (Figure 3C). In general, female mice kept at 23°C have more UCP1 protein than male mice housed at the same temperature.

Female mice had much higher BAT *Bmp8b* expression than male mice, and ovariectomy almost completely ablated BAT *Bmp8b* expression (Figure 3D). The effects of the cold challenge on BAT *Bmp8b* expression in sham-operated male mice was comparable to what we found with intact males: an approximately 44-fold induction (*p* < 0.025). Cold exposure also elevated BAT *Bmp8b* expression in sham-operated female mice, but with an approximately 17-fold induction, the cold effect was less strong than that in male mice. This different relative effect of cold on BAT *Bmp8b* expression between the sexes can be explained by the already higher BAT *Bmp8b* expression in female mice compared to male mice. As in the previous experiment, BAT *Fgf1* expression was lower in female mice than that in male mice (*p* < 0.025) while ovariectomy resulted in elevated BAT *Fgf1* expression (Figure 3D). Cold exposure resulted in a similar and in general higher BAT *Fgf1* expression in all four groups of mice. Thus, cold exposure induces BAT *Bmp8b* and *Fgf1* expression independent of sex and presence of gonads.

### DES induces BAT *Bmp8b* expression *in vivo* but not *in vitro*

**Figure 4:**
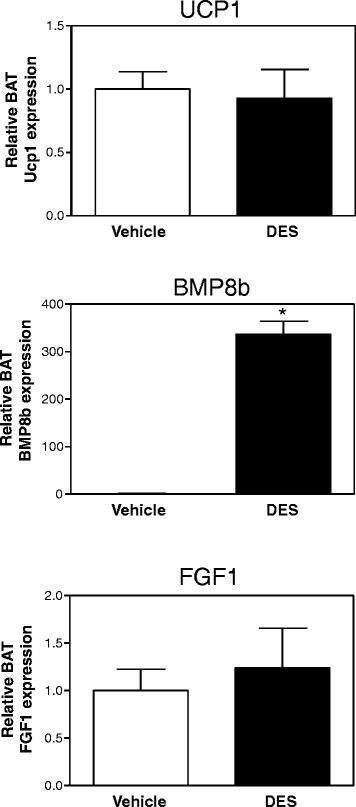
**DES treatment induces BAT** ***Bmp8b*** **expression in male mice.** *Ucp1*, *Bmp8b*, and *Fgf1* mRNA expression in BAT of male mice injected with diethylstilbestrol (DES) or olive oil vehicle for 1 week. Results were normalized to 18S ribosomal RNA (*Rn18s*) and beta-2 microglobulin (*B2m*) with data from placebo or vehicle mice defined as ‘1’. Values are averages ± SEM; *n* = 6; *p < 0.05 vs*.* vehicle (Mann–Whitney *U* test).

**Figure 5:**
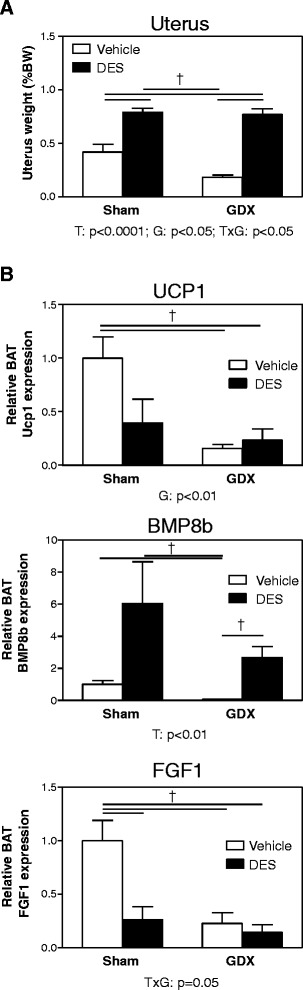
**DES treatment increases BAT** ***Bmp8b*** **expression in female mice. (A)** Uterus weight as percent of body weight (BW) of female mice 2 weeks following gonadectomy (GDX) or sham operation and exposure to daily injections with diethylstilbestrol (DES) or olive oil vehicle during the last week. **(B)** *Ucp1*, *Bmp8b*, and *Fgf1* mRNA expression in BAT of female mice 2 weeks following gonadectomy (GDX) or sham operation and exposure to daily injections with diethylstilbestrol (DES) or olive oil vehicle during the last week. Results were normalized to 18S ribosomal RNA (*Rn18s*) and beta-2 microglobulin (*B2m*) with data from placebo or vehicle mice defined as ‘1’. Values are averages ± SEM; *n* = 6; †*p* < 0.025 (Mann–Whitney *U post hoc* test when significance was detected by two-way ANOVA). Depicted below the graphs are the significant *p* values of the two-way ANOVA tests for either treatment (T), GDX (G), or the interaction between treatment and GDX (TxG).

Since estrogens are mainly produced by the ovaries and are only present at lower concentrations in males, the data so far suggest that they are obvious candidate hormones controlling *Bmp8b* and *Fgf1* expression in BAT. To test whether estrogens indeed regulate BAT *Bmp8b* and *Fgf1* expression *in vivo*, male mice, sham-operated female mice, and ovariectomized female mice were injected with the stable E2 analogue DES. In male mice, those injected with DES significantly gained more weight than the control mice (10.2% ± 1.5% vs*.* 1.2% ± 0.5%, DES vs*.* control, *p* < 0.05, Mann–Whitney *U* test) and showed a strongly induced BAT *Bmp8b* expression (Figure 4). However, DES did not affect BAT *Fgf1* expression. In both sham-operated and ovariectomized female mice, 1 week DES treatment also significantly increased body weight (Table 2). DES had strong estrogenic effects since it induced uterus weight by 1.9-fold in the sham-operated female mice and by 4.2-fold in ovariectomized female mice (Figure 5A). Interestingly, 2 weeks after ovariectomy, BAT *Ucp1* expression was severely reduced but this was not corrected by DES (Figure 5B). Ovariectomy also reduced BAT *Bmp8b* and *Fgf1* mRNA concentrations. DES treatment enhanced *Bmp8b* expression in sham-operated and ovariectomized female mice by 504% ± 261% and 3,472% ± 970%, respectively. In addition, DES reduced BAT *Fgf1* expression by 74% in sham-operated females.

**Table 2:** **Effect of GDX and DES treatment on body weight of female mice**

	**Sham**		**GDX**		**Two-way ANOVA results**		
	**Vehicle**	**DES**	**Vehicle**	**DES**	**Surgery**	**Treatment**	**Interaction**
BW before surgery	19.8 ± 0.4	19.6 ± 0.4	19.9 ± 0.6	19.8 ± 0.4	NS	NS	NS
BW 1 week after surgery	20.2 ± 0.5	20.1 ± 0.4	21.6 ± 0.3	21.4 ± 0.5	*p* < 0.005	NS	NS
Change in BW (%) 1 week after surgery	2.1 ± 0.5	3.0 ± 1.3	8.9 ± 2.5	8.3 ± 2.7	*p* < 0.01	NS	NS
BW after 1 week treatment	21.3 ± 0.5	23.0 ± 0.4	23.2 ± 0.2	24.1 ± 0.4	*p* < 0.005	*p* < 0.005	NS
Change in BW (%) 1 week after treatment	5.8 ± 1.0	14.3 ± 1.6	7.5 ± 1.3	12.7 ± 1.2	NS	*p* < 0.0001	NS

Female mice received gonadectomy (GDX) or a sham operation. After 1 week, mice were injected daily with diethylstilbestrol (DES) or olive oil vehicle for 1 week. Values are averages ± SEM; *n* = 6.

NS, not significant.

**Figure 6:**
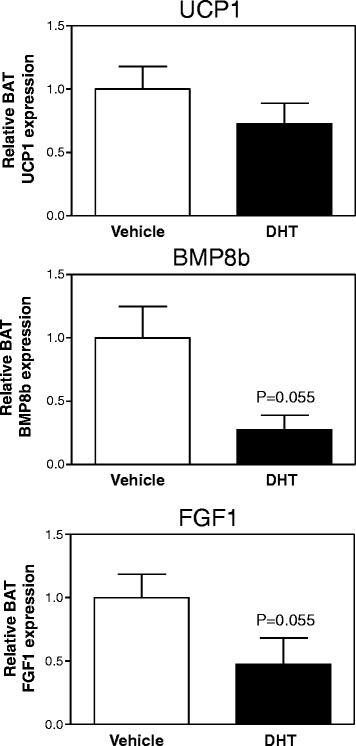
**One week DHT treatment reduces BAT** ***Bmp8b*** **expression in female mice.** *Ucp1*, *Bmp8b*, and *Fgf1* mRNA expression in BAT of female mice who received daily injections with dihydrotestosterone (DHT) for 1 week. Results were normalized to 18S ribosomal RNA (*Rn18s*) and beta-2 microglobulin (*B2m*) with data from vehicle mice defined as ‘1’. Values are averages ± SEM; *n* = 6; *p < 0.05 vs*.* vehicle (Mann–Whitney *U* test).

**Figure 7:**
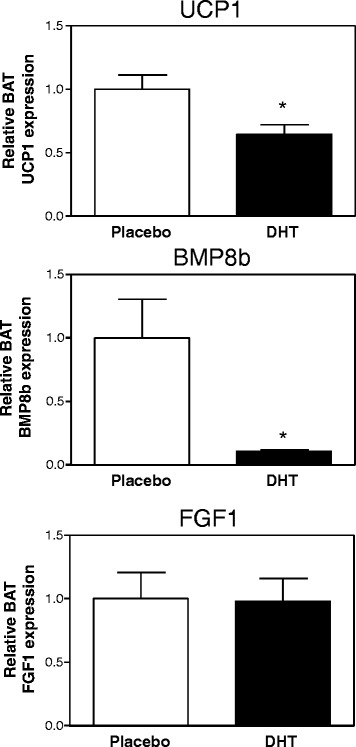
**Long-term effects of DHT treatment on BAT gene expression in female mice.** *Ucp1*, *Bmp8b*, and *Fgf1* mRNA expression in BAT of female mice treated with a DHT or placebo pellet for 90 days. Results were normalized to 18S ribosomal RNA (*Rn18s*) and beta-2 microglobulin (*B2m*) with data from placebo mice defined as ‘1’. Values are averages ± SEM; *n* = 6; *p < 0.05 vs*.* placebo (Mann–Whitney *U* test).

To decrease estrogen concentrations in female mice via another mechanism than removal of the ovaries, we injected intact female mice with DHT for 1 week, which resulted in a tendency towards higher body weights (induction of 8.4% ± 1.0% vs*.* 5.0% ± 1.0%, DHT vs*.* control, *p* = 0.065, Mann–Whitney *U* test). One week DHT treatment resulted in a tendency towards reduced BAT *Bmp8b* and *Fgf1* expression, albeit not significant (Figure 6). However, the once daily DHT injections were not sufficient to constantly suppress the release of gonadotropin-releasing hormone (GnRH) by the hypothalamus since uterus weights were only marginally affected by DHT (data not shown). When female mice were treated with DHT for a much longer period of 90 days, they were approximately 21% heavier than vehicle-treated mice as described previously [24], but also had a severe reduction of BAT *Bmp8b* expression while BAT *Fgf1* expression was not affected (Figure 7). Altogether, these data clearly show that estrogens regulate BAT *Bmp8b* expression *in vivo*.

**Figure 8:**
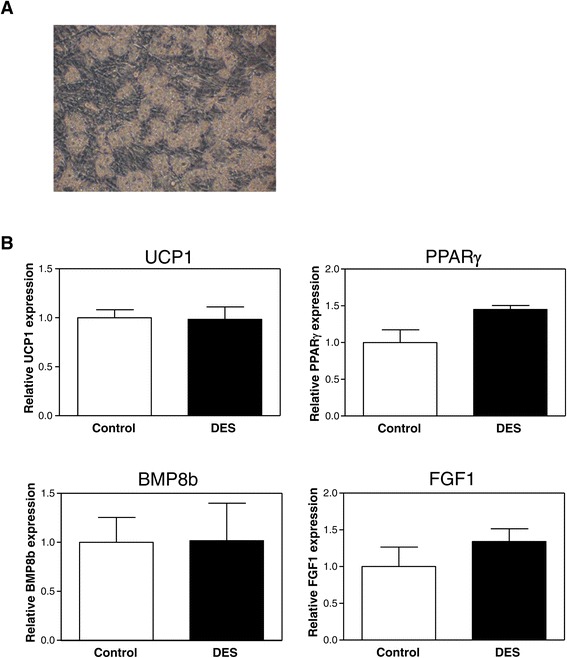
**Effect of DES treatment on brown adipocyte gene expression** ***in vitro*** **.** Primary cultured murine brown adipocytes were treated with or without 10 uM diethylstilbestrol (DES) for 24 h. **(A)** Representative picture of the primary murine brown adipocyte culture after 12 days of differentiation. **(B)** *Ucp1*, *Pparγ*, *Bmp8b*, and *Fgf1* mRNA expression in primary brown adipocytes. Expression was normalized to hypoxanthine-guanine phosphoribosyltransferase (*Hprt*) with data from control adipocytes defined as ‘1’. Values are averages ± SEM; *n* = 4; *p < 0.05 (Mann-Whiney *U* test).

Next, to investigate whether the effects *in vivo* on BAT *Bmp8b* expression are due to direct effects of DES on the brown adipocytes, we treated cultured primary brown adipocytes with DES (Figure 8). As reported before for the 3 T3-L1 pre-adipocyte cell line [27], DES induced the expression of the gene encoding the lipogenic transcription factor proliferator-activated receptor gamma (PPARγ) by 45%, although this failed to reach statistical significance. Since DES did not affect *Bmp8b* expression in brown adipocytes *in vitro*, the effect of DES on BAT *Bmp8b* expression *in vivo* are likely mediated via an indirect mechanism, presumably via the hypothalamus.

## Discussion

The presence of UCP1 in the mitochondrial membranes allows BAT to produce heat instead of ATP upon oxidation of fatty acids. Since BAT has very high potential to oxidize large amounts of lipids from the system, BAT is an attractive target tissue to combat obesity. However, despite renewed interest in this tissue after the discovery of BAT in adult humans [2-4], no specific therapeutics to activate BAT have been identified yet. Catecholamines and thyroid hormone T_4_ are known to induce BAT activity [1], but systemic administration of these compounds results in severe side effects such as hypertension and tachycardia. Detailed investigation of paracrine and autocrine factors, such as BMPs and FGFs, that mediate BAT activity might tell us how to pursue the hunt for novel BAT activators. Studies have shown that female mice have more active BAT than male mice and that estrogens might be key hormones in these differences [6,7,12,14,15]. Therefore, we also compared the sex differences in BAT in mice with or without gonads, thus with or without their sex steroid hormones, using the physiologically most relevant method to activate BAT *in vivo*: cold exposure [28]. In summary, we found that expression of the gene encoding BMP8b in the BAT depot is upregulated by both cold exposure and the presence of ovaries. Additional experiments in which mice had received DES and female mice that had a changed estrogen/androgen ratio to a male-like nature showed that increased estrogen/androgen ratios result in a higher BAT *Bmp8b* mRNA expression *in vivo*.

It has been proposed that females have more active BAT than males [6,7,12,14,15], and one would expect that female mice also have higher BAT *Ucp1* mRNA expression and protein content. In our hands, however, BAT *Ucp1* mRNA expression did not differ between male and female mice (Figures 2B and 3C). In contrast, we found that, compared to male mice, female mice have more BAT UCP1 protein than male mice (Figure 3D), which is the protein involved in thermogenesis [29]. Of interest, the 45-day period after ovariectomy did not affect BAT *Ucp1* mRNA expression (Figures 2B and 3C), but when mice were analyzed only 2 weeks after ovariectomy, BAT *Ucp1* mRNA expression was significantly reduced (Figure 5B). Thus, a direct effect of ovariectomy is indeed reduced BAT *Ucp1* mRNA expression, but a longer period of ovariectomy results in a compensatory effect on *Ucp1* mRNA expression. How this latter mechanism is regulated is, to our knowledge, not known.

Since recent and older literature shows that E2 induces BAT *Ucp1* mRNA expression [7,12,13,15], our findings that 1-week daily injections with DES did not significantly affect the expression of *Ucp1* in BAT (Figures 4 and 5B) is unexpected. DES has a similar affinity for the estrogen receptor α (ERα) as E2 albeit fivefold more potent [30]; thus, similar effects of DES and E2 on mRNA expression profiles are to be expected. A plausible explanation for the differences between our DES study and previous studies might be the method of delivery. While most research groups implanted estrogen-containing pellets, we give daily DES injections and collected tissue samples 24 h after the last injection. Since the clearance of DES in pregnant mice is very rapid during the first 30 min, although slowed downed after 1 h [31], it is possible that analysis of *Ucp1* mRNA expression 24 h after the last injection is too late to find any effects. Unfortunately, we are unaware of studies on DES kinetics in murine BAT.

The failure of DES to induce *Ucp1* mRNA expression despite its effects on BAT metabolism via ERα might also be the result of reduced estrogen-receptor-related receptor (ERR) α, ERRβ, and/or ERRγ transcriptional activity. Tremblay et al*.* [32] found that DES inhibits transcriptional activity of all three ERR subtypes in trophoblast stem cells. Other studies have shown that both ERRα and ERRβ mediate *Ucp1* expression in brown adipocytes [33,34]. However, whether DES also inhibits transcriptional activity of ERR subtypes and hence reduces *Ucp1* mRNA expression in (brown) adipocytes *in vivo* needs to be confirmed by additional experiments.

Our results suggest that the observed higher expression of *Bmp8b* in female mice may be the result of regulation by estrogens, since ovariectomy abolished BAT *Bmp8b* expression (Figure 2C) while DES administration to intact male mice, sham-operated female mice, and ovariectomized female mice induced BAT *Bmp8b* expression (Figures 4 and 5B). In addition, we show that changing the estrogen/androgen ratio to a male-like nature by short-term or long-term treatment of female mice with DHT also negatively affected BAT *Bmp8b* expression (Figures 6 and 7). As such, *Bmp8b* may be a direct target of estrogen action via ERα and/or ERβ in brown adipocytes. However, additional *in vitro* experiments with cultured brown adipocytes showed that DES had no direct effect on *Bmp8b* mRNA expression in these cells (Figure 8). Thus, it is more likely that DES indirectly mediates BAT *Bmp8b* expression. For instance, DES may act on ERα located in the ventromedial nucleus of the hypothalamus that will result in an inhibition of AMP-activated protein kinase (AMPK), subsequently leading to induction of BAT activation, as recently shown for E2 [15], and cause *Bmp8b* expression to rise.

Of interest, BAT *Bmp8b* expression did not correlate with changes in body weight in our experiments. Both DHT and DES treatment resulted in elevated body weights, but DHT treatment reduced whereas DES treatment induced BAT *Bmp8b* expression. The increase in body weight upon DES treatment has been reported before and has been attributed to enhance expansion of the WAT depot [27]. Altogether, our findings underscore that changing the estrogen/androgen ratio *per se* affects BAT *Bmp8b* expression, irrespective of effects on body weight.

Our studies also showed an induction of the gene encoding for FGF1 in cold-exposed mice (Figure 1C). FGF1 has recently been shown to be crucial for the hyperplastic effects of PPARγ agonists in adipose tissue depots which most importantly also involves angiogenesis [35]. Of interest, PPARγ is a nuclear receptor that is also required for brown adipocyte differentiation [36]. Effects of FGF1 on brown adipocytes themselves are conflicting. On the one hand, FGF1 has been reported to upregulate *Ucp1* mRNA expression [23] while others found that FGF1 downregulated expression of the gene encoding lipoprotein lipase (LPL) [37]. This latter observation is counterintuitive since cold exposure increases BAT *Lpl* mRNA expression [38] and LPL is thought to be crucial in the uptake of the fatty acid substrates by activated BAT. Hence, the precise roles of FGF1 in BAT remain to be elucidated.

The finding that a member of the BMP family and a member of the FGF family are expressed in a sex-dependent manner in BAT is intriguing. The interaction between the signaling pathways of various BMP and FGF family members is well known; a strong balance between both pathways is important in several developmental processes [39]. For instance, BMP and FGF ligands have opposing effects in cardiomyocyte differentiation [40], apical ectodermal ridge and hindbrain development [41,42], and oligodendrocyte precursors generation [43]. However, it remains to be determined whether such a crosstalk exists between FGF1 and BMP8b signaling in BAT.

The data collected in this article are generated from mouse experiments leaving the translational question open. Mejhert et al*.* [44] mapped the presence of FGFs in human WAT and found that only *FGF1*, *FGF2*, *FGF7*, *FGF9*, and *FGF18* were present in human WAT. They almost exclusively used female subjects, making comparisons between the sexes not possible. The same study showed that only FGF1 was released by the white adipocytes but this secretion did not contribute to FGF1 in the circulation, suggesting again that FGF1 very likely has a paracrine and/or autocrine function. Another study found that human WAT from obese men expresses *FGF1*, *FGF2*, *FGF7*, *FGF9*, *FGF10*, and *FGF18* [45]. *FGF1* and *FGF9* were more abundantly expressed in omental WAT. Since this WAT depot is considered less likely to gain BAT-like properties, the presence of *FGF1* and *FGF9* in omental WAT is rather unexpected since expression of *Fgf1* and *Fgf9* was induced in activated murine BAT (Figure 1C). In mice, *Bmp8b* expression responded strongly to cold exposure and also showed a large sex-differential expression pattern. However, it is unknown whether human BAT expresses *BMP8b*. One of the BMP type I receptors (BMPR1A) is expressed in human WAT [46], but no sex-differences have been reported yet.

## Conclusions

In conclusion, we discovered that BAT *Bmp8b* expression is regulated by presence of ovaries and ERα ligands such as DES in mice.

## Abbreviations

AMPK: AMP-activated protein kinase
BAT: brown adipose tissue
BMP: bone morphogenetic protein
DES: diethylstilbestrol
DHT: dihydrotestosterone
E2: estradiol
ERα: estrogen receptor alpha
ERR: estrogen-receptor-related receptor
^18^F-FDG: ^18^F-deoxyglucose
FGF: fibroblast growth factor
GDX: gonadectomy
GnRH: gonadotropin-releasing hormone
NE: norepinephrine
LPL: lipoprotein lipase
PET: positron emission tomography
PPARγ: proliferator-activated receptor gamma
TG: triglyceride
UCP1: uncoupling protein 1
WAT: white adipose tissue
